# Deep Learning-Driven Pose Extraction and Spatiotemporal Refinement for Human Detection and Distance Estimation on Railway Tracks with Thermal Imaging

**DOI:** 10.3390/s26165080

**Published:** 2026-08-11

**Authors:** Milan Pavlović, Ivan Ćirić, Danijela Ristić Durrant, Miloš Simonović, Mladen Kuzev, Lubomir Dimitrov, Vlastimir Nikolić

**Affiliations:** 1 The Academy of Technical and Preschool Studies Niš, Beogradska 18, 18000 Niš, Serbia; 2 Faculty of Mechanical Engineering, University of Niš, Aleksandra Medvedeva 14, 18000 Niš, Serbia; ivan.ciric@masfak.ni.ac.rs (I.Ć.); danijela.ristic.durrant@masfak.ni.ac.rs (D.R.D.); milos.simonovic@masfak.ni.ac.rs (M.S.); mladenkuzev@gmail.com (M.K.); vlastimir.nikolic@masfak.ni.ac.rs (V.N.); 3 Faculty of Mechanical Engineering, Technical University Sofia, Bul. Sv. Kliment Ohridski 8, 1756 Sofia, Bulgaria; lubomir_dimitrov@tu-sofia.bg

**Keywords:** thermal imaging, human detection, railway safety, pose extraction, distance estimation

## Abstract

Human presence in rail track areas represents a critical safety risk, particularly under low-visibility conditions where RGB-based monitoring systems become unreliable. This paper presents a thermal-only railway monitoring framework for human detection, auxiliary pose-based skeletal representation, identity-preserving tracking, temporal refinement, track zone classification, and camera-to-human distance estimation. The proposed approach combines a YOLO-based human detector trained on annotated thermal railway images with a pretrained YOLO pose model used as an auxiliary module to obtain a reduced 13-keypoint skeletal representation for thermal-image interpretation. ByteTrack is used for identity association across frames, while Kalman filtering reduces frame-to-frame keypoint localization jitter and supports short-term trajectory continuity during temporary missed detections. Unlike fully learned monocular depth estimation, the proposed distance estimation method exploits rail track geometry as a physical scene reference. A YOLO-based rail track detection model extracts the rail region, after which rail line candidates are fitted, and the apparent rail track width is measured at the vertical position of the detected human. Distance is estimated using an inverse-perspective relationship based on the known railway gauge. Experiments on thermal video sequences acquired along the Niš–Prokuplje railway line demonstrate reliable human detection, stable tracking, effective track zone classification, and distance estimation consistent with real distance trends.

## 1. Introduction

According to the International Union of Railways (UIC), total significant accidents increased by 21.5% between 2020 and 2024, reaching a five-year high of 2018 accidents in 2024 [1]. In addition, level crossing accidents increased by 16% in 2024 [1]. In Europe, trespassing incidents account for 65.6% of all railway-related fatalities, making unauthorized presence in the rail track area the leading cause of rail-related deaths [2]. These statistics underscore the critical need for reliable real-time early human-detection systems capable of operating under poor visibility conditions, which represents the primary motivation for the proposed approach.

In recent years, computer vision and deep learning techniques have been increasingly applied to railway monitoring systems, particularly for object and human detection. Under good visibility conditions (daylight, clear weather), modern object detection models using RGB camera inputs and deep convolutional neural networks, particularly YOLO-based architectures, enable accurate localization and classification of objects in real time, making them suitable for intelligent surveillance systems [3,4].

However, real-world railway environments frequently involve low-light and low-visibility conditions, including nighttime operation, tunnels, fog, and adverse weather. In such conditions, RGB cameras may fail to capture sufficient contrast, leading to missed detections and false alarms. As a result, reliable human detection becomes challenging, limiting the effectiveness of standard vision-based safety systems. To address these limitations, thermal imaging has emerged as a promising alternative, enabling detection regardless of illumination conditions [5,6]. Consequently, several studies have demonstrated that thermal imaging significantly improves pedestrian detection performance in complex scenarios where RGB-based approaches fail due to insufficient illumination and reduced contrast [7,8]. Nevertheless, thermal imagery introduces challenges such as lower spatial resolution, reduced structural detail, and increased noise, which can negatively impact detection accuracy and feature extraction. In addition to detection, accurate estimation of the distance between the detected human and the monitoring system is of critical importance for risk assessment and decision-making processes.

Motivated by these challenges, this paper proposes a thermal-only framework for human detection, auxiliary pose-based skeletal representation, identity-preserving tracking, temporal refinement, and distance estimation under low-visibility conditions. The proposed approach combines a YOLO-based human detector trained on annotated thermal railway images, a pretrained YOLO pose model used to obtain an auxiliary skeletal representation, ByteTrack-based identity association, and Kalman-based temporal refinement to improve output stability across consecutive frames. In addition, a separate YOLO-based rail track detection model is used to extract rail track geometry, while the camera-to-human distance is estimated geometrically from the apparent rail track width at the vertical position of the detected human. By exploiting the known railway gauge as a physical scene reference, the proposed framework provides a distance estimation procedure tailored to fixed-camera, straight-track thermal railway scenarios.

## 2. Related Work

Vision-based perception for railway safety has attracted increasing attention due to the need for early detection of obstacles, pedestrians, and unauthorized presence in railway areas. Compared with road traffic perception, railway perception is constrained by long stopping distances, limited maneuverability, fixed infrastructure geometry, and the need for reliable long-range detection. Ristić-Durrant et al. [9] reviewed vision-based on-board obstacle detection and distance estimation for railways and emphasized that environmental perception in rail applications remains insufficiently developed, particularly under low-visibility and long-range conditions. Public rail-scene datasets such as RailSem19 [10] have also contributed to the development of semantic understanding in railway environments by providing rail-relevant annotations including rails and scene objects. RailSem19 is a public dataset for semantic rail-scene understanding with 8500 annotated railway/tram scenes, which illustrates the growing interest in rail-specific computer vision resources. Chen et al. introduced RailFOD23, a dataset for foreign object detection on railroad transmission lines, containing 14,615 images with 40,541 annotated objects. The dataset focuses on foreign objects affecting railroad power transmission infrastructure and demonstrates the use of synthetic data generation and image augmentation to address the scarcity of abnormal railway scenarios [11]. Rampriya et al. introduced UAV-RSOD, an unmanned aerial vehicle captured dataset for railroad segmentation and obstacle detection. UAV-RSOD contains 315 raw UAV images, labeled and masked data for railroad semantic segmentation, and augmented annotated images for obstacle detection covering several obstacle classes, including person, boulder, barrel, branch, jerry can, and iron rod [12]. A sparse-to-dense guided fusion framework for railway three-dimensional object detection uses synchronized camera and point-cloud data from an experimental maglev inspection scenario [13]. The framework extracts structural anchors from sparse point-cloud features, retrieves image features around valid geometric locations to reduce redundant dense computation, and applies dynamic cross-modal attention to balance visual and geometric cues. In addition, Yang et al. proposed Graph-MDETR, a graph-guided Mamba-DETR network for UAV-based catenary support component detection in electrified railways. Their method introduces a DAMamba-Adapter backbone and graph-guided modules to improve the detection of multi-scale, multi-category, and occluded catenary support components in UAV images [14]. However, such datasets are primarily oriented toward RGB rail-scene understanding rather than thermal human detection and distance estimation in safety-critical low-visibility scenarios.

Deep learning-based object detection has been widely adopted for transportation and surveillance applications because of its ability to localize objects in real time. YOLO-based detectors, from the original YOLO formulation [3] to later versions such as YOLOv4 [4], have been extensively used in intelligent monitoring systems due to their favorable accuracy–speed ratio. Rahman et al. investigated the applicability of deep neural networks to railway obstacle detection by comparing several transfer-learning models, including MobileNetV2, YOLOv5, ResNet50, VGG19, and VGG16, on a custom railway-track obstacle dataset [15]. However, most approaches are RGB-based and therefore strongly dependent on illumination, contrast, and weather conditions. This dependence limits their applicability in low-visibility scenarios such as nighttime, tunnels, fog, and other environmental and weather conditions. Therefore, while RGB detectors are effective under favorable conditions, they do not fully address the operational requirements of thermal railway monitoring.

Thermal imaging provides an important alternative because it captures emitted radiation rather than reflected visible light, making it suitable for nighttime and poor illumination conditions. Liu et al. [6] proposed a thermal infrared vehicle and pedestrian detection method for complex scenes and reported improved detection performance by adapting YOLOv3-based detection components to thermal imagery. You et al. [5] addressed thermal pedestrian detection for edge computing devices, highlighting the need for efficient thermal detection under practical deployment constraints. Nevertheless, thermal imagery introduces its own limitations, including lower spatial resolution, weaker texture, blurred object boundaries, reduced anatomical detail, and increased ambiguity at long distances. These limitations are particularly important for railway applications, where people may appear as small thermal objects far from the camera.

Multispectral pedestrian detection has been proposed to combine the complementary strengths of visible and thermal modalities. König et al. [16] proposed a fully convolutional region proposal network for multispectral human detection by combining visible and thermal image information. Although their method is not railway-specific and does not address distance estimation, it demonstrates the value of visible–thermal fusion for robust human detection under challenging visibility conditions. The KAIST multispectral pedestrian benchmark provides aligned RGB–thermal image pairs and has become a widely used benchmark for all-day pedestrian detection [17]. Cross-modal and fusion-based methods have shown that combining visible and thermal information can improve robustness under changing illumination conditions [18,19]. However, multispectral systems require synchronized and calibrated sensors, increasing system complexity and deployment cost.

Human pose estimation has become a central task in computer vision because skeletal keypoints provide structural information beyond bounding boxes. Cao et al. introduced real-time multi-person 2D pose estimation using part affinity fields [20], while HRNet [21] improved keypoint localization by maintaining high-resolution representations throughout the network. YOLO-based pose estimation has also been introduced as a heatmap-free and efficient approach for multi-person pose estimation [22], and modern YOLO pose implementations are commonly pretrained on COCO keypoints. However, most pose estimation methods are developed and evaluated primarily on RGB datasets. Their direct use on thermal images is problematic because thermal imagery often lacks facial details, fine contours, and reliable limb boundaries. Ultralytics YOLO pose models are commonly trained on the COCO keypoints dataset, and the default YOLO pose representation uses 17 human keypoints corresponding to different body joints [23].

There are few studies that have investigated pose or posture estimation in infrared or thermal imagery. Guo et al. [24] introduced an infrared human posture-related task that lies between detection and full pose estimation, indicating that infrared imagery can support structural human representation but also requires task-specific adaptation. Recent work on human pose estimation using thermal images [25] further demonstrates the feasibility of thermal pose analysis, while emphasizing the need for domain-specific adaptation due to the lack of texture, reduced anatomical detail, and limited availability of annotated thermal pose datasets.

Tracking-by-detection methods are commonly used to preserve object identities across video frames. SORT [26] demonstrated that simple Kalman filtering combined with Hungarian assignment can provide fast online multi-object tracking, while DeepSORT [27] improved identity preservation by incorporating appearance-based association. ByteTrack [28] improved tracking by associating not only high-confidence detections but also low-confidence detections that may still correspond to true objects. This is particularly relevant for thermal railway monitoring, where detection confidence can decrease due to low contrast, long distance, or partial occlusion. Temporal refinement is also important because thermal detections and keypoints may fluctuate from frame to frame. Kalman filtering has long been used for prediction and smoothing in tracking systems, including SORT [26].

In railway applications, human detection must be complemented by distance estimation, since operational decisions depend not only on the detection of a human, but also on the distance between the detected human and the monitoring system and the available time for warning or intervention. General monocular depth estimation methods attempt to infer scene depth from a single image using learned representations [29,30]. Such methods can be powerful, but they often require large datasets and may generalize poorly across camera setups, environments, and imaging modalities. In railway applications, previous work has investigated monocular obstacle distance estimation using RGB and thermal imagery. Haseeb et al. [31] proposed a DisNet-based approach for long-range obstacle detection and distance estimation from monocular camera images in railway scenes. Ćirić et al. [32] investigated AI-powered obstacle distance estimation for autonomous train operation, while Pavlović et al. [7] performed distance estimation between the camera and detected objects using a homography-based method and validated against real measured distances. More recently, the FuzzyH method [33] combined homography-based distance estimation with fuzzy logic for railway scenarios. These studies demonstrate the feasibility of thermal and vision-based railway distance estimation, but they do not fully integrate human detection, skeletal representation, tracking, temporal refinement, and rail track-guided geometric distance estimation in a single framework.

While RGB and multispectral approaches can provide rich visual information, they require sufficient illumination or additional synchronized sensors, which increases system complexity and limits their applicability in low-visibility railway monitoring. Many railway perception studies focus on obstacle detection or scene understanding, but do not explicitly address human pose representation and temporal refinement in thermal railway imagery. Existing thermal pedestrian detection studies mostly focus on bounding-box detection, while structural skeleton representation is less frequently considered. Furthermore, tracking and temporal filtering are often treated as separate additions rather than integrated components of a railway safety monitoring framework. In addition, distance estimation is often addressed either through fully learned monocular depth models or as a separate geometric procedure, without direct integration with human tracking and railway scene geometry.

The proposed framework addresses these limitations by combining a trained YOLO-based human detector, a pretrained YOLO pose model used as an auxiliary skeletal representation, ByteTrack-based identity association, Kalman-based temporal refinement, YOLO-based rail track detection, and rail track-guided distance estimation. By exploiting the known railway gauge as a physical scene reference, the proposed method provides a transparent distance estimation procedure tailored to fixed-camera, straight-track thermal railway monitoring scenarios.

## 3. Methodology

**Figure 1:**
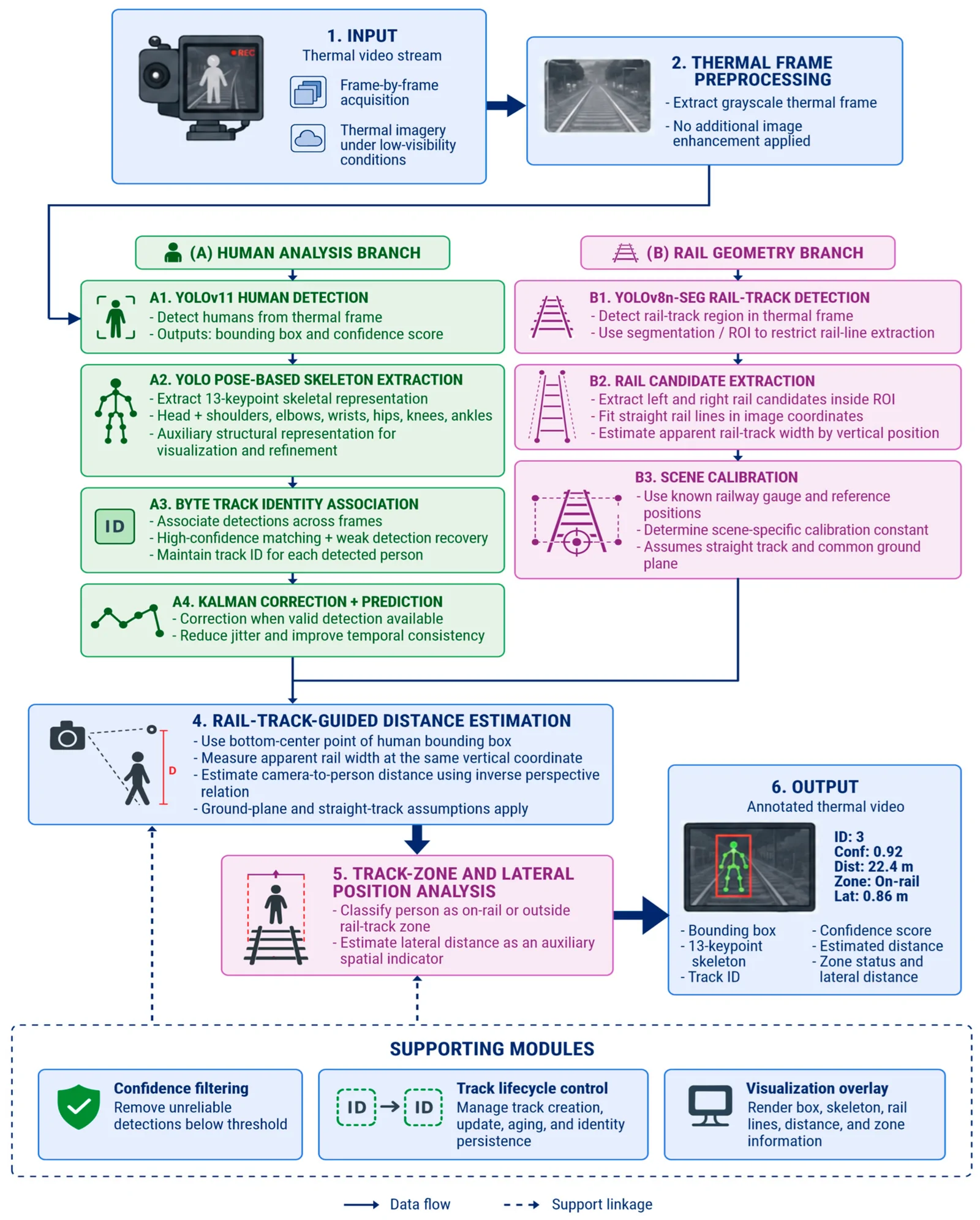
Methodology flowchart of the proposed system.

The proposed framework is designed for human detection, auxiliary pose-based skeletal representation, temporal refinement, tracking, and distance estimation in thermal railway imagery acquired under low-visibility conditions. The processing chain consists of six main stages: thermal frame preprocessing, YOLO-based human detection, YOLO pose-based skeleton extraction, multi-object tracking and spatiotemporal refinement, rail track detection, and rail track-guided distance estimation (Figure 1).

Unlike direct monocular depth estimation, the proposed method does not estimate distance only from learned visual features. Instead, the distance between the camera and each detected human is calculated using the perspective geometry of the rail track. YOLO-based models are used to detect humans and the rail track, while the final distance is obtained geometrically from the apparent rail track width at the vertical position of the detected human.

Human detection is performed using a YOLO11s-based object detection model trained on annotated thermal railway images. The dataset contained 339 manually annotated thermal images with one object class, human, where human instances were labeled using bounding boxes. Due to the relatively small size of the available annotated dataset, an 80–10–10 split was adopted to preserve a sufficiently large training subset while still providing separate validation and test subsets. Accordingly, 271 images were used for training, 34 images for validation, and 34 images for testing. The YOLO11s model was initialized from pretrained weights and then fine-tuned on the collected thermal railway dataset. The model was trained with an input image size of 1024 pixels and a batch size of 4. Automatic optimizer selection was used, and RandAugment-based automatic augmentation was applied during training. Although the training was initially configured for 80 epochs, it was stopped after 27 epochs, when the validation performance had stabilized. Since the automatic optimizer setting was used, the learning-rate configuration was selected automatically by the Ultralytics training framework rather than manually specified.

**Figure 2:**
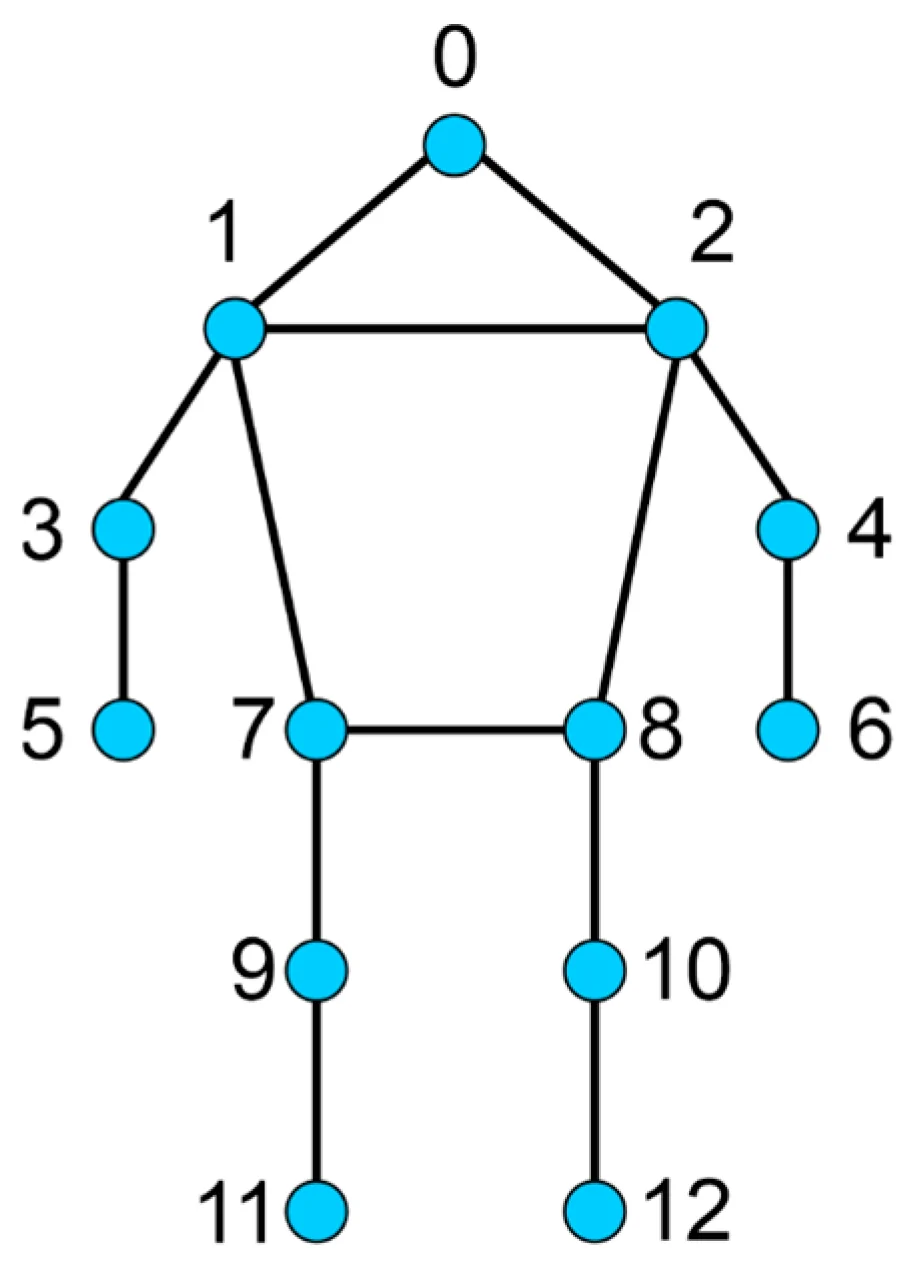
The 13 keypoints of the skeleton.

In addition to bounding box-based human detection, a YOLO26x-pose model was used to extract auxiliary skeletal information for detected humans. Unlike the YOLO11s human detector and the rail track segmentation model, the pose model was not additionally trained or fine-tuned on the collected thermal railway dataset. Instead, a pretrained YOLO pose model was used directly to obtain skeletal keypoints. Therefore, the keypoints on thermal images were not manually annotated or independently verified as ground-truth pose labels. The pose-estimation model is not presented as an independently validated thermal pose-estimation method, but as an auxiliary skeletal-representation module that supports visual interpretation of human posture and temporal behavior. Standard COCO-based pose models represent the human body using 17 keypoints, including five facial/head-related keypoints corresponding to the nose, eyes, and ears, as well as the main body joints. However, the reduction from the default COCO-based 17-keypoint representation to the adopted 13-keypoint representation was motivated by the limited spatial resolution and reduced anatomical detail of thermal imagery. Wang et al. have shown that low-resolution conditions significantly affect the reliability of human pose estimation, especially for small or weakly visible anatomical details [34]. In long-range thermal railway scenes, facial keypoints are often difficult to distinguish reliably because distant humans occupy only a small number of pixels. The adopted reduced representation included: left and right shoulders (1 and 2), left and right elbows (3 and 4), left and right wrists (5 and 6), left and right hips (7 and 8), left and right knees (9 and 10), and left and right ankles (11 and 12). The head point (0) was obtained by averaging the coordinates of the available facial keypoints (nose, eyes, and ears) detected by the pose model (Figure 2). In this way, facial keypoints were represented by a single head keypoint. This provides a compact skeletal representation suitable for visual interpretation and temporal representation in low-detail thermal imagery.

The rail track segmentation model was based on YOLOv8n-seg and was trained on manually annotated thermal railway images prepared in Roboflow and exported in YOLO format. The rail track dataset contained 285 manually annotated thermal images. Due to the relatively small size of the available annotated dataset, an 80–10–10 split was adopted in order to preserve a sufficiently large training subset while still providing separate validation and test subsets. Accordingly, 228 images were used for training, 29 images for validation, and 28 images for testing. The YOLOv8n-seg model was initialized and trained with an input image size of 1024 pixels and a batch size of 16. Automatic optimizer selection was used, with lr0/lrf values of 0.01/0.01. The applied augmentation settings included Mosaic = 1.0, Translate = 0.1, Scale = 0.5, Fliplr = 0.5, and Erasing = 0.4. The model was trained for 41 epochs. Since the dataset was relatively limited in size, broader generalization to more diverse railway environments requires additional validation on larger annotated thermal datasets.

During inference, the human detector used a confidence threshold of 0.44 and an IoU threshold of 0.40 for non-maximum suppression. The pose model used a confidence threshold of 0.18, while the rail track segmentation model used a confidence threshold of 0.60. Human detection was performed at an inference resolution of 640 pixels, whereas rail track segmentation was performed at 512 pixels.

For identity association, ByteTrack was used with the following parameters: track_high_thresh = 0.44, track_low_thresh = 0.05, new_track_thresh = 0.60, track_buffer = 300, and match_thresh = 0.80. Temporal refinement was performed using Kalman filtering. The KalmanRail filter used process and measurement noise values of 10.0 and 45.0, respectively. The KalmanBox filter used process and measurement noise values of 0.1 and 0.1, while the KalmanPose filter used values of 0.05 and 0.1, respectively.

The implementation was developed in Python using the Ultralytics framework. Model inference was performed on Google Colab using an NVIDIA T4 GPU.

Let the input thermal video sequence be defined as:

(1) $$V=\left\{I_{1},I_{2},...,I_{T}\right\}\;,$$

where *I_t_* denotes the thermal frame at time *t* and *T* is the total number of frames. Each frame was represented as grayscale image, and no additional image enhancement was applied.

For each input frame *I_t_*, the human detection model outputs a set of detected humans:

(2) $$D_{t}=\left\{d_{1}^{t},d_{2}^{t},...,d_{N}^{t}\right\}\;,$$

where *N* is the number of detected humans. Each detection is represented as:

(3) $$d_{i}^{t}=\left\{B_{i}^{t},c_{i}^{t}\right\}\;,$$

where $B_{i}^{t}$ is the bounding box of the detected human and $c_{i}^{t}$ is the detection confidence score.

If the bounding box is represented by upper-left and lower-right image coordinates, it is defined as:

(4) $$B_{i}^{t}=\left(x_{min,i}^{t},y_{min,i}^{t},x_{max,i}^{t},y_{max,i}^{t}\right)\;.$$

Since the human detector was trained on a limited number of annotated thermal images, it was used for human localization in the analyzed railway scenarios, while broader generalization requires additional validation on larger and more diverse datasets.

In addition to bounding box-based human detection, a YOLO26x-pose model is used to extract skeletal information for detected humans. Unlike the human detection and rail track detection models, the pose model was not additionally trained on the collected thermal railway dataset. Instead, a pretrained YOLO pose model was used to obtain skeletal keypoints.

The pose output was used as an auxiliary structural representation of detected humans. As described above, the pretrained YOLO26x-pose output was reduced to the adopted 13-keypoint representation in order to obtain a compact skeletal description suitable for low-detail thermal imagery.

The keypoint set is defined as:

(5) $$K_{i}^{t}=\left\{k_{i,1}^{t},k_{i,2}^{t},...,k_{i,13}^{t}\right\}\;,$$

where each keypoint is represented by its image coordinates and confidence score:

(6) $$k_{i,j}^{t}=\left(u_{i,j}^{t},v_{i,j}^{t},s_{i,j}^{t}\right)\;.$$

The pose output was used as an auxiliary structural representation of detected humans, while the primary localization for distance estimation was based on the bottom-center point of the human bounding box.

After human detection, ByteTrack was used as a tracking-by-detection method to associate detections across consecutive frames and preserve the identity of each detected human. In the first association stage, detections with confidence scores above 0.44 were treated as high-confidence detections. Detections with confidence scores above 0.05 were retained for the second association stage in order to recover weak detections caused by low thermal contrast, long distance, or partial occlusion. New tracks were initialized only when the detection confidence exceeded 0.60. The matching threshold was set to 0.80, and detection scores were fused with the association cost.

The Intersection over Union between bounding boxes is used as the main spatial association criterion:

(7) $$IoU\left(B_{a},B_{b}\right)=\frac{B_{a}\cap B_{b}}{B_{a}\cup B_{b}}\;.$$

Detections satisfying predefined confidence and matching thresholds are assigned to existing tracks, while unmatched detections initialize new tracks.

Thermal video sequences may contain temporal instability caused by weak object boundaries, reduced thermal contrast, sensor noise, and temporary missed detections. Therefore, Kalman filtering is applied to reduce frame-to-frame keypoint localization jitter and to support short-term trajectory continuity. For each tracked human, the bounding box center is represented by a constant-velocity state:

(8) $$s_{i}^{t}=[x_{i}^{t},y_{i}^{t},v_{x,i}^{t},v_{y,i}^{t}{]}^{T}\;,$$

where $x_{i}^{t}$ and $y_{i}^{t}$ denote the bounding box center, while $v_{x,i}^{t}$ and $v_{y,i}^{t}$ denote the corresponding velocity components. For pose refinement, the same constant-velocity Kalman filtering principle was applied independently to the image coordinates of each skeletal keypoint in order to reduce frame-to-frame keypoint localization jitter.

The prediction step is performed at every frame, while the correction step is performed only when a valid detection is available. If a human is temporarily missed, the previous track is propagated for a limited number of frames. If the human is not detected for more than $M_{max}$ consecutive frames, the track is terminated. In this study, $M_{max}$ was set to 70 frames, corresponding to approximately 10 s. Kalman filtering is used only for temporal refinement. It should be noted that track_buffer = 300 is an internal ByteTrack parameter used during identity association, whereas $M_{max}$ = 70 is an additional implementation-level limit used in the proposed framework for short-term track propagation during temporary missed detections. It does not replace the detector and does not generate new detections independently.

Distance estimation relies on the extraction of rail track geometry from thermal images. For this purpose, a separate YOLOv8n-seg-based rail track detection model, denoted as $S_{MODEL}$, was trained with one class, rail track. The rail track detection model was trained using 285 manually annotated thermal images prepared in Roboflow and exported in YOLO format. The purpose of this model is not to estimate distance directly, but to detect the rail track region and support the extraction of left and right rail geometry. The rail track region was annotated using polygon mask. After detection, the rail track bounding box was used to restrict the region of interest in which left and right rail line candidates were extracted. Since the rail track model was trained on a limited number of annotated images, it was used as a scene-geometry extraction module for the analyzed experimental scenarios, rather than as a general-purpose railway segmentation model.

The distance between the thermal camera and each detected human is estimated using the perspective geometry of the rail track. The method is based on the fact that the apparent width of the rail track in the image decreases as the distance from the camera increases. Therefore, the rail track is used as a scene-based geometric reference for distance estimation.

The detected rail track region is used to extract left and right rail line candidates. A straight line is fitted to each rail line candidate in the image coordinate system. Since the vertical image coordinate increases downward, the left and right rails are represented as functions of the vertical coordinate:

(9) $$X_{L}\left(Y\right)=m_{L}Y+c_{L}\;,$$

(10) $$X_{R}\left(Y\right)=m_{R}Y+c_{R},$$

where $X_{L}(Y)$ and $X_{R}(Y)$ denote the horizontal positions of the left and right rail at the vertical image coordinate Y, while $m_{L}$, $m_{R}$, $c_{L}$, and $c_{R}$ are the corresponding line parameters.

The vertical coordinate of the vanishing point, corresponding to the apparent intersection of the two rail lines, is calculated as:

(11) $$Y_{horizon}=\frac{c_{R}-c_{L}}{m_{L}-m_{R}}\;.$$

For each detected human, the point used for distance estimation is defined as the bottom-center point of the human bounding box. This point is selected as an approximation of the human’s ground-contact point. If the bounding box is represented by its upper-left and lower-right image coordinates:

(12) $$B_{i}^{t}=\left(x_{min,i}^{t},y_{min,i}^{t},x_{max,i}^{t},y_{max,i}^{t}\right)\;,$$

then the bottom-center point is calculated as:

(13) $$P_{bc,i}^{t}=\left(\frac{x_{min,i}^{t}+x_{max,i}^{t}}{2},y_{max,i}^{t}\right)\;,$$

where $P_{bc,i}^{t}$ represents the image approximation of the ground-contact point of human i in frame t. The vertical coordinate used for distance estimation is therefore:

(14) $$Y_{bc,i}^{t}=y_{max,i}^{t}\;.$$

At this vertical coordinate, the apparent rail track width in pixels is calculated as:

(15) $$w_{px}(Y_{bc,i}^{t})=X_{R}(Y_{bc,i}^{t})-X_{L}\left(Y_{bc,i}^{t}\right),$$

or equivalently:

(16) $$w_{px}\left(Y_{bc,i}^{t}\right)=\left(m_{R}-m_{L}\right)Y_{bc,i}^{t}+\left(c_{R}-c_{L}\right)\;.$$

The physical distance is then estimated using an inverse perspective relationship inspired by the pinhole camera model. The real railway gauge is assumed to be $W_{real}=1.435\;m$, which corresponds to the standard railway gauge of 1435 mm. The estimated distance is calculated as:

(17) $${\hat{D}}_{i}^{t}=\frac{W_{real}\cdot K_{cal}}{w_{px}(Y_{bc,i}^{t})}\;,$$

where ${\hat{D}}_{i}^{t}$ is the estimated distance of human i in frame t, $W_{real}$ is the real railway gauge, $w_{px}(Y_{bc,i}^{t})$ is the apparent rail track width in pixels at the vertical position of the detected human, and $K_{cal}$ is a scene-specific calibration constant expressed in pixels.

The calibration constant $K_{cal}$ was determined experimentally using three reference positions with known distances from the camera. For each reference position, the apparent rail track width in pixels was measured at the vertical image coordinate corresponding to the bottom-center point of the human bounding box.

For each reference distance, the calibration constant was calculated as:

(18) $$K_{cal,i}=\frac{D_{ref,i}\cdot w_{px,i}}{W_{real}}\;,$$

where $D_{ref,i}$ is the known reference distance, $w_{px,i}$ is the apparent rail track width in pixels measured at that distance and $W_{real}=1.435\;m$ is the real railway gauge (Table 1). The arithmetic mean of these values was approximately 6481 px and was rounded to the scene-specific calibration constant $K_{cal}=6500\;px$. Therefore, the final distance estimation equation becomes:

(19) $${\hat{D}}_{i}^{t}=\frac{9327.5}{w_{px}(Y_{bc,i}^{t})}\;.$$

This formulation reflects the inverse relationship between distance and apparent rail track width. As a human moves farther from the camera, the bottom-center point of the bounding box approaches the vanishing point, the apparent width of the rail track decreases, and the estimated distance increases.

The method assumes that the detected human is located on or in close vicinity of the railway ground plane and that the visible railway segment can be approximated by two straight converging rail lines.

In addition to estimating the camera-to-human distance, the proposed system evaluates the lateral position of each detected human with respect to the rail tracks. This step provides additional safety-relevant information because a human located between the rail tracks represents a higher-risk situation than a human located outside the track area.

The track zone classification uses the bottom-center point of the human bounding box and the fitted left and right rail track-line geometry defined in the previous subsection. For each detected human, the bottom-center point is compared with the horizontal positions of the left and right rail tracks at the same vertical image coordinate. If this point lies between the two rail lines, the detected human is classified as being inside the track zone. In the output visualization, the corresponding track zone status $Z_{i}^{t}$ is displayed as ON RAIL, the bounding box is shown in red, and a warning message is generated.

If the bottom-center point is located outside the rail boundaries, the lateral pixel distance between this point and the nearest fitted rail track $d_{lat,i}^{t}$ is calculated. For a human located to the left of the track, the distance is calculated with respect to the left rail, while for a human located to the right of the track, the distance is calculated with respect to the right rail. This pixel distance is then converted into meters using the local pixel-to-meter scale derived from the apparent rail track width and the known railway gauge, as defined in the rail track-guided distance estimation procedure.

This output distinguishes between humans located inside and outside the track zone, while also providing the lateral distance from the nearest rail line when the human is outside the track zone. Since independent ground-truth measurements of lateral distance were not available in the present experiments, the estimated lateral distance is interpreted as an auxiliary spatial indicator rather than as a fully validated distance measurement. The accuracy of this indicator depends on the quality of human bounding box localization, the reliability of rail lines fitting, and the assumption that the detected human is located approximately on the same ground plane as the rail track.

For each processed frame, the final system output is defined as:

(20) $$O_{t}=\{{\tilde{B}}_{i}^{t},{\tilde{K}}_{i}^{t},ID_{i}^{t},c_{i}^{t},{\hat{D}}_{i}^{t},Z_{i}^{t},d_{lat,i}^{t}{\}}_{i=1}^{N_{t}}\;,$$

where ${\tilde{B}}_{i}^{t}$ is the temporally refined bounding box, ${\tilde{K}}_{i}^{t}$ is the skeletal representation obtained from the YOLO pose model, $ID_{i}^{t}$ is the assigned tracking identity, $c_{i}^{t}$ is the detection confidence score, ${\hat{D}}_{i}^{t}$ is the estimated distance from the camera, $Z_{i}^{t}$ is the track zone status, and $d_{lat,i}^{t}$ is the estimated lateral distance from the nearest rail line when the detected human is outside the track zone.

The output video displays detected humans with bounding boxes, skeletal keypoints, tracking identities, confidence scores, estimated camera-to-human distances, track zone status, and, when applicable, lateral distance from the nearest rail line. This output provides an interpretable representation of human presence, movement, and proximity in railway environments under low-visibility conditions.

## 4. Results and Discussion

### 4.1. Experimental Setup

**Figure 3:**
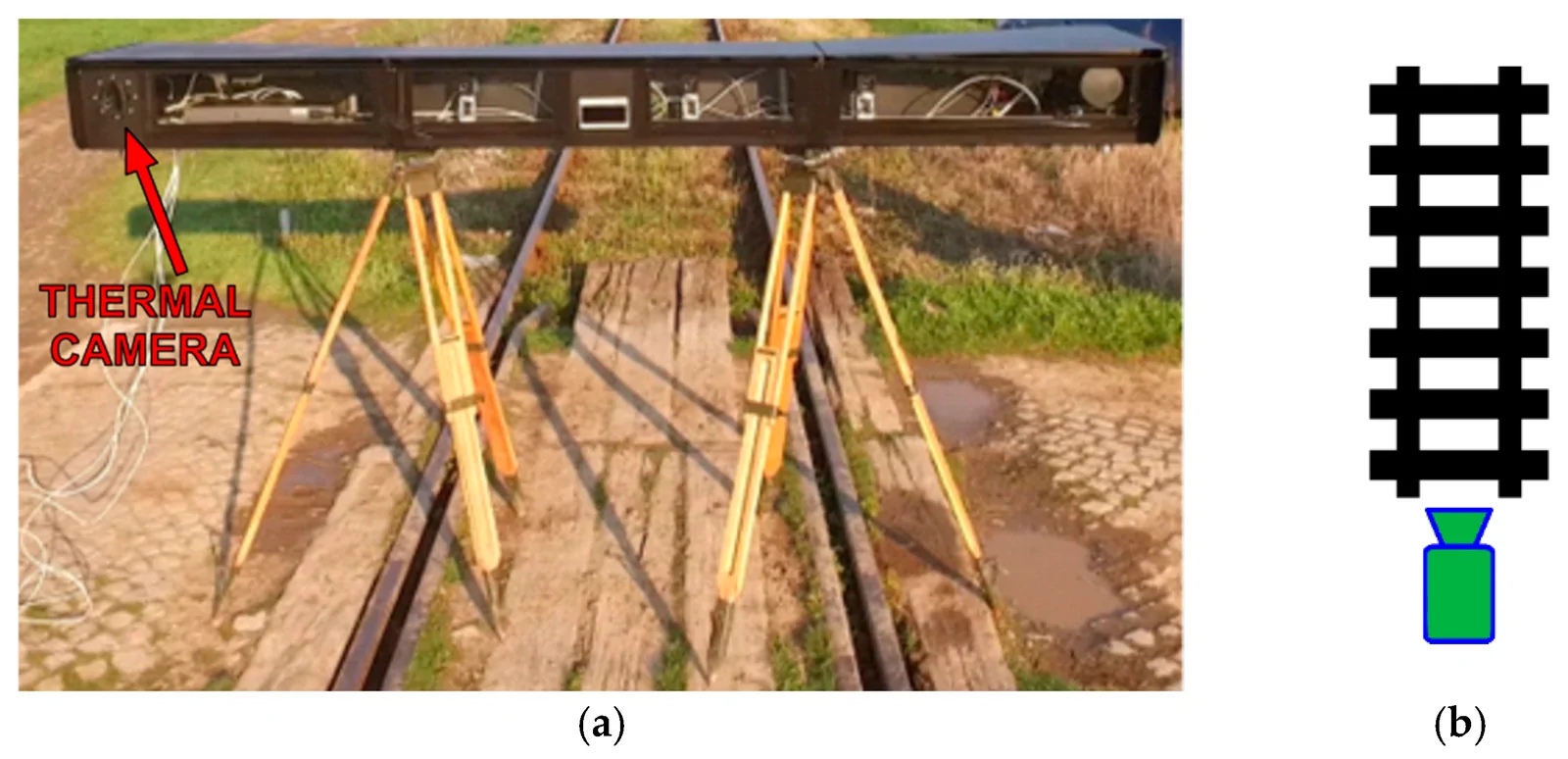
(**a**) Experimental setup on field [35]. (**b**) Thermal camera position during experiments.

The experimental setup consists of a multimodal sensing platform deployed in real railway conditions, including three monocular RGB cameras, one thermal camera, one night vision system, and a laser scanner. All sensors are mounted in a rigid custom-designed metal housing to ensure mechanical stability and protection under field conditions (Figure 3a). In this study, only the thermal camera data were used for training and evaluation. The remaining sensors were not used in the detection, pose, tracking, or distance estimation results.

The experiments were conducted at multiple railway test sites in Serbia along the Niš–Prokuplje railway, with approval granted by the Serbian railway authorities for experimental use. All human participants involved in the experiments were authorized volunteers informed about the purpose and procedure of the experiments before participation. All safety procedures were followed to ensure that no participant was exposed to operational railway traffic or uncontrolled railway-track conditions. During experiments, a FLIR TAU2 thermal camera with a resolution of 640 ×  480 pixels and focal length of 100 mm was used. The camera was fixed and positioned centrally between rail lines at a height of 1.5 m above the ground corresponding to a feasible mounting position on a locomotive. The optical axis of the camera was oriented parallel to the direction of rail tracks, providing a symmetric field of view across both rails (Figure 3b). The mounting height and orientation are fixed throughout all experiments to ensure consistent geometric conditions for distance estimation. All test scenarios included humans located both on and alongside the rail track at different distances from the thermal camera, as well as moving along rail tracks, enabling evaluation of detection, tracking, and distance estimation performance under realistic conditions. Real distances from camera to humans were measured manually using a measuring tape in order to avoid the uncertainty associated with GPS-based measurements. The real distance was defined as the distance from the thermal camera position to the human’s ground-contact position along the railway track.

The experimental validation was conducted using four representative thermal video scenarios: a stationary two-human scenario, a quasi-static local-movement scenario, a dynamic zig-zag short-range scenario, and a three-human dynamic long-range scenario. The images used for training the YOLO-based human detection and rail track segmentation models were not reused as test images. The reference distances of 30 m, 90 m, and 150 m were used only to determine the scene-specific calibration constant $K_{cal}$. The validation of the distance-estimation procedure was performed using additional measured distances that were not used for calibration. When the same distances appeared in the validation material, they corresponded to different frames and were not reused for deriving $K_{cal}$.

### 4.2. Human Detection and Distance Estimation

Before evaluating the complete framework in railway video sequences, the main perception and temporal-refinement components were quantitatively assessed. During training, the YOLO11s human detection model achieved its best validation-stage performance near the end of training, with a validation precision of 0.979 and a validation mAP@0.5 of 0.990 at epoch 26. Similarly, the YOLOv8n-seg rail track segmentation model achieved a maximum validation mAP@0.5 of 0.946 during training, while the final validation mAP@0.5 was 0.930. The effect of Kalman-based temporal refinement was evaluated using an inter-frame keypoint localization jitter metric. Instead of manually annotating sub-pixel reference keypoint positions in consecutive thermal frames, which is prone to human error and bias, the evaluation was performed in a stationary scenario in which the tracked persons remained physically still. Under this condition, the expected true displacement of each skeletal keypoint between consecutive frames can be considered approximately zero; therefore, any measured inter-frame displacement can be interpreted as localization jitter. The jitter error was calculated as the Euclidean distance between the coordinates of the same keypoint of the same tracked person in two consecutive frames. Due to the limited availability of continuous stationary frames, the evaluation was restricted to a continuous sequence of 36 frames, corresponding to 35 inter-frame displacement intervals, and included two tracked persons and all 13 skeletal keypoints used in the proposed representation. For human ID 1, the strongest stabilization was observed for the head keypoint, where the raw inter-frame jitter decreased from 0.253 px/frame to 0.114 px/frame after Kalman filtering, corresponding to a reduction of 54.9%. When all 13 keypoints were considered, the average full-body jitter for human ID 1 decreased from 0.916 px/frame to 0.691 px/frame, corresponding to a reduction of 24.6%. For human ID 2, the average full-body jitter decreased from 0.303 px/frame to 0.230 px/frame, corresponding to a reduction of 24.1%. These results show that Kalman filtering reduced frame-to-frame keypoint jitter and improved the temporal stability of the skeletal representation in the analyzed stationary sequence. Overall, these results indicate that the individual components provide a basis for the subsequent video-level evaluation of human detection, rail track extraction, identity-preserving tracking, temporal refinement, track zone classification, and distance estimation.

**Figure 4:**
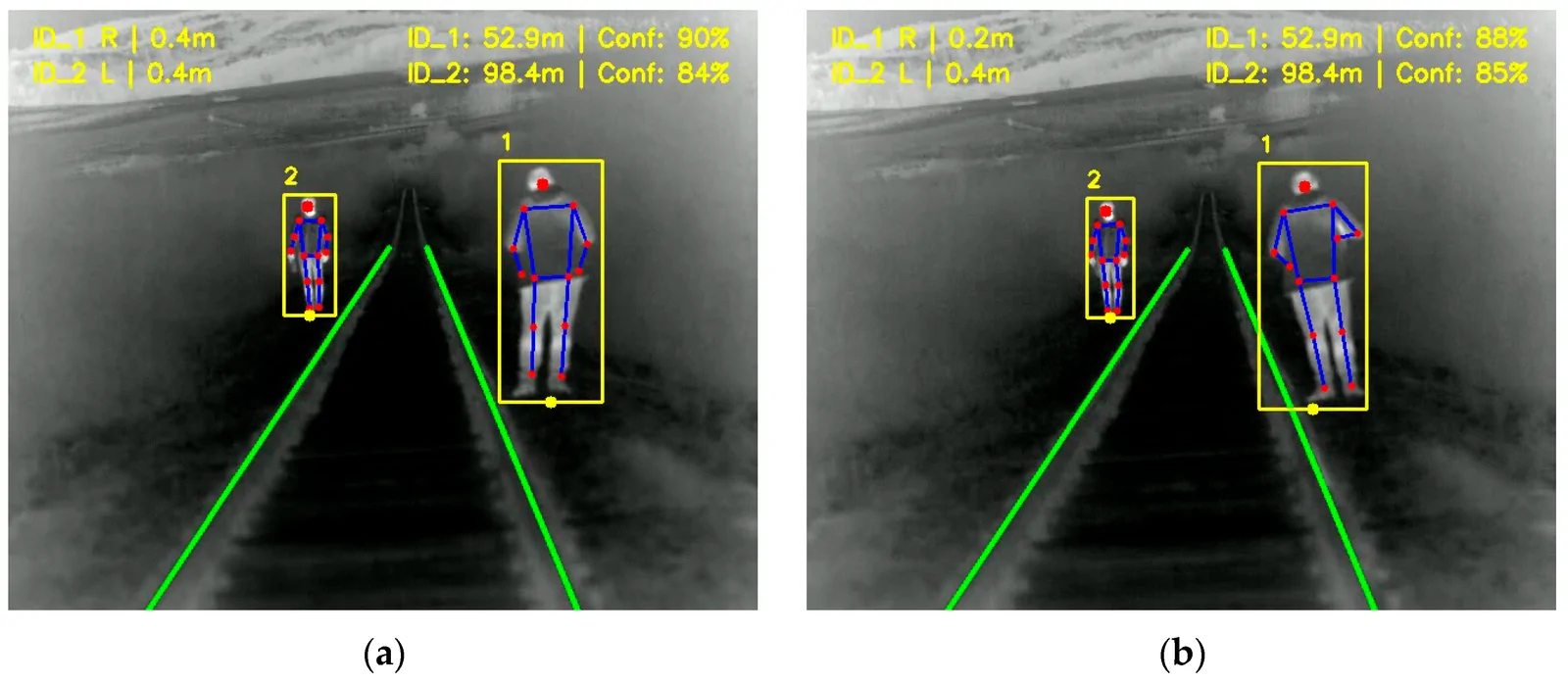
Results of first evaluation of proposed system: (**a**) stationary scenario, (**b**) quasi-static scenario. The displayed outputs include rail track detection (marked in green), bounding boxes (marked in yellow), identity labels (marked in yellow), track zone status with position of detected human with respect to the rail lines (L—left, R—right) and estimated lateral distance from the nearest rail line when the detected human is outside the track zone (marked in yellow), confidence scores (marked in yellow), estimated camera-to-human distances (marked in yellow), auxiliary skeletal representations (marked in blue links with red keypoints).

The performance of the proposed system was first evaluated in a controlled scenario involving two humans positioned at 50 m and 100 m from the experimental setup. During the experiment, both humans remained stationary in close vicinity of the rail tracks in order to test the capability of the system (Figure 4a). The results demonstrated that both humans were successfully detected and localized, with each human clearly identified by a bounding box and a corresponding 13-keypoint skeletal representation. The detection confidence for the more distant human was greater than 80%, while for the closer human it was greater than 85%. In addition, the estimated distances were 52.9 m for the closer human and 98.4 m for the more distant human, closely matching the ground truth values with errors of 5.8% and 1.6%, respectively (Figure 4a). These results indicate that the proposed system can provide consistent human detection and distance estimation in this controlled moderate-distance scenario.

However, in order to examine whether small lateral movements and changes in body posture affect human detection, auxiliary skeletal visualization, tracking continuity, and distance estimation when the longitudinal camera-to-human distance remains approximately constant, the second controlled scenario was performed. In this scenario, the same camera setup and real distances as in the previous experiment were used. The scenario can therefore be considered as quasi-static because humans were allowed to move locally in the vicinity of the railway track without approaching or moving away from the camera (Figure 4b). The results shown in Table 2 indicate that the system maintained consistent detection performance under these conditions, with no significant degradation in localization accuracy, pose estimation stability, confidence levels, or distance estimation. This indicates that the proposed framework is not limited to fully stationary humans, although this scenario should be interpreted as a local movement stability test rather than a full dynamic distance-tracking evaluation.

A dynamic zig-zag short-range scenario was performed to evaluate the robustness of the system when one human remains inside the track zone while another human moves laterally and longitudinally near the rail track. In this scenario, one human was positioned between the rail lines at a real distance of 150 m from the camera, while the second human moved in a zig-zag trajectory from approximately 100 m toward the camera. This setup was used to examine human detection, auxiliary skeletal representation, tracking, camera-to-human distance estimation, and track zone classification under local dynamic movement.

**Figure 5:**
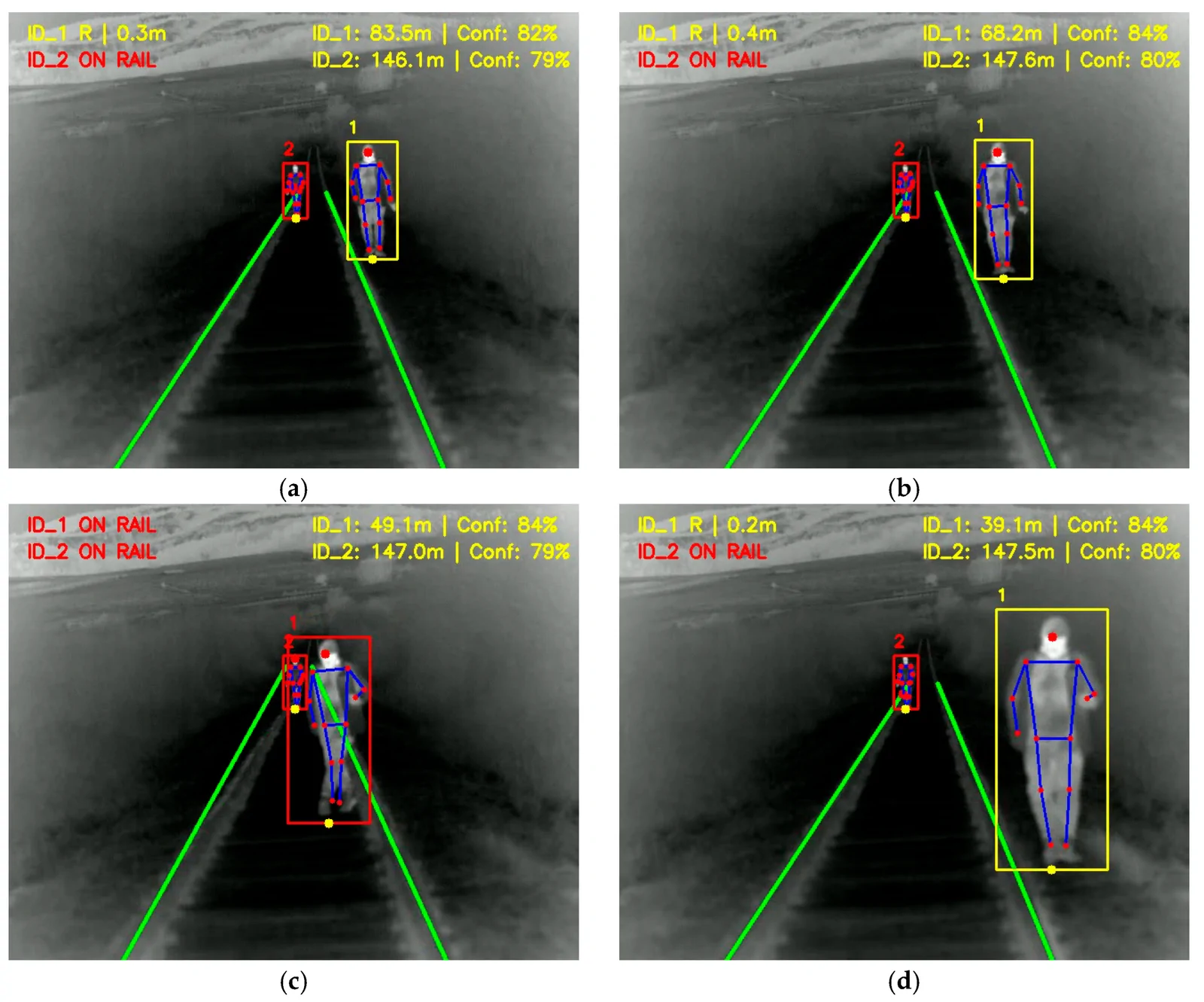
Results of dynamic zig-zag short-range scenario. The moving human is shown at measured reference distances of (**a**) 90 m, (**b**) 70 m, (**c**) 50 m, and (**d**) 40 m, while the stationary human remains positioned at 150 m inside the track zone. The displayed outputs include rail track detection (marked in green), bounding boxes, identity labels (marked in yellow), track zone status with position of detected human with respect to the rail lines (L—left, R—right) and estimated lateral distance from the nearest rail line when the detected human is outside the track zone (marked in yellow), confidence scores (marked in yellow), estimated camera-to-human distances (marked in yellow), auxiliary skeletal representations (marked in blue links with red keypoints). Yellow bounding boxes and yellow track zone statuses indicate humans classified as being outside the track zone, whereas red bounding boxes indicate humans classified as being inside the track zone with displayed red ON RAIL track zone status.

The stationary human was correctly classified as being inside the track zone and displayed with the ON RAIL warning status, and the corresponding bounding box and warning status were displayed in red. For the stationary human positioned at 150 m inside the track zone, the estimated distance varied between 146.1 m and 147.6 m, resulting in an absolute error of 2.4–3.9 m and a relative error of approximately 1.6–2.6%. The detection confidence for the stationary human remained stable, despite the relatively larger distance from the camera, with values of 79%, 80%, 79%, and 80%. For the moving human, the largest error occurred at the longest analyzed distance, where the estimated distance was 83.5 m instead of the measured reference distance of 90 m (Figure 5a), corresponding to an absolute error of 6.5 m and a relative error of 7.2%. At shorter distances, the errors were considerably lower, 1.8 m at 70 m, 0.9 m at 50 m, and 0.9 m at 40 m (Figure 5b–d), corresponding to relative errors of approximately 2.6%, 1.8%, and 2.3%, respectively. The corresponding track zone classification indicated that the human was located to the right of the rail track at 90 m and 70 m, then entered the track zone at 50 m and was displayed with the ON RAIL warning status and moved outside the track zone again on the right side at 40 m. The detection confidence for the moving human remained high, with values of 82%, 84%, 84%, and 84%, respectively.

The correct classification of the stationary human as being inside the track zone, together with the ON RAIL warning status and red bounding box, indicates that the system can identify the most safety-critical spatial condition in the analyzed scenario. For the moving human, the system consistently followed changes in lateral position, correctly distinguishing between positions outside the track zone and temporary entry into the track zone. The confidence scores remained stable for both humans, indicating that local movement and changes in position did not noticeably affect human detection in this scenario. The distance-estimation results further suggest that the method is more accurate at shorter distances, while the larger error observed at 90 m indicates increased sensitivity at longer ranges, where small variations in rail line fitting and bounding box localization have a greater influence on the estimated distance (Table 3). Thus, the results support the feasibility of the proposed framework for short-range dynamic monitoring, while also highlighting the need for careful interpretation of distance estimates as the camera-to-human distance increases.

In order to further evaluate the robustness of the proposed system over a wide range of distances, an additional experiment was conducted. A three-human dynamic scenario was performed in which three humans simultaneously moved from long-range distances toward the camera. In this experiment, the humans moved from approximately 400 m toward the experimental setup, while their position with respect to the track zone changed during motion. The purpose of this scenario was to examine whether the system can maintain human detection, bounding-box localization, track zone classification, distance estimation, and auxiliary skeleton visualization in a more complex multi-human railway scene. The system was evaluated over the entire distance range; however, for clarity, several representative checkpoints were selected and reported in Table 4 in order to illustrate the system behavior at characteristic distances and track zone configurations.

**Figure 6:**
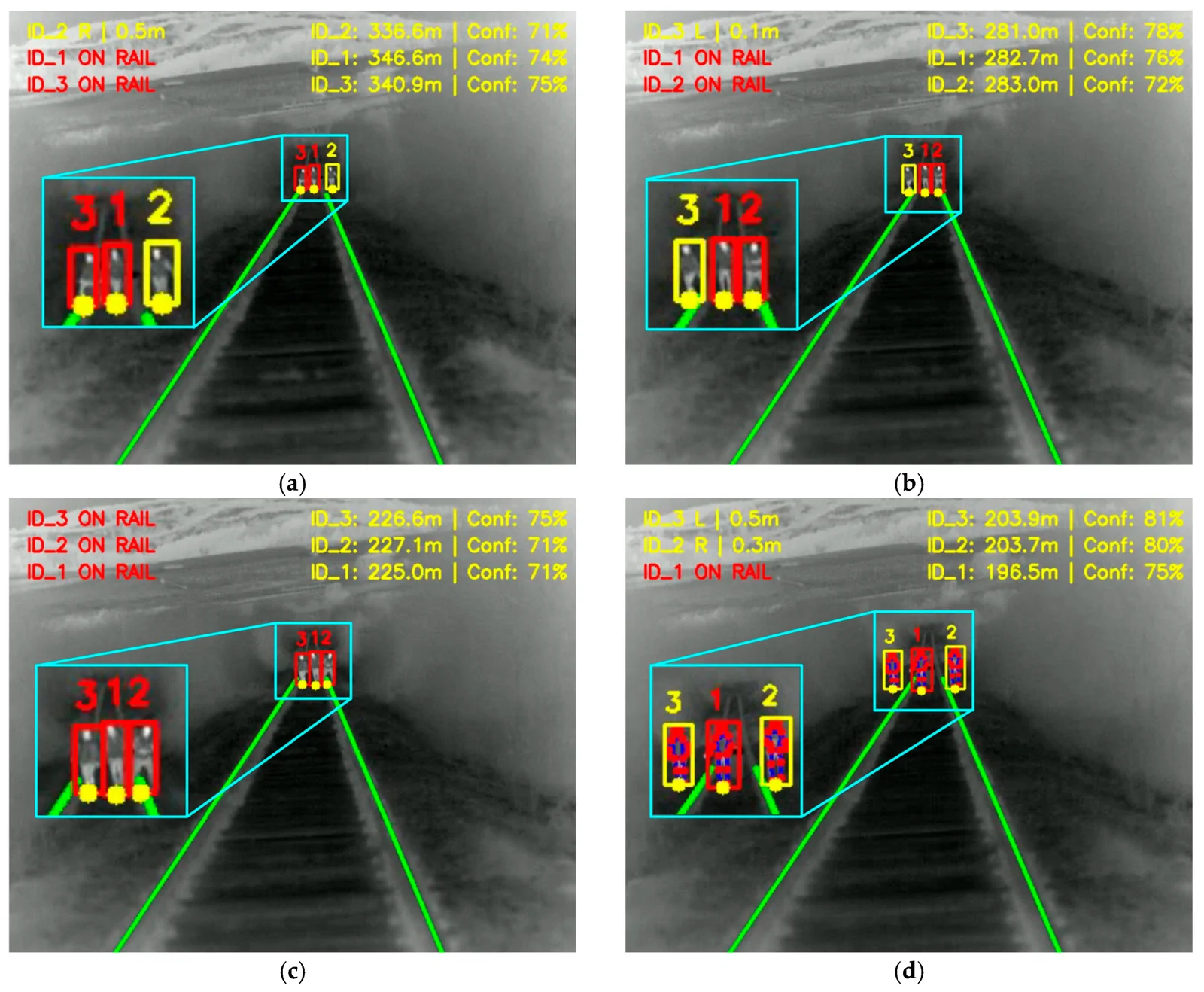
Results in three-human dynamic scenario in the range of approximately 400–200 m, with zoomed-in views marked in cyan. The three humans are shown at measured reference distances of approximately (**a**) 370 m, (**b**) 300 m, (**c**) 250 m, and (**d**) 200 m from the thermal camera. The displayed outputs include rail track detection (marked in green), bounding boxes, identity labels (marked in yellow), track zone status with position of detected human with respect to the rail lines (L—left, R—right) and estimated lateral distance from the nearest rail line when the detected human is outside the track zone (marked in yellow), confidence scores (marked in yellow), estimated camera-to-human distances (marked in yellow), auxiliary skeletal representations (marked in blue links with red keypoints). Yellow bounding boxes and yellow track zone statuses indicate humans classified as being outside the track zone, whereas red bounding boxes indicate humans classified as being inside the track zone with displayed red ON RAIL track zone status.

At larger distances, from approximately 400 m to 200 m, the system successfully detected all three humans, generated bounding boxes, estimated their camera-to-human distances, and provided track zone status. The auxiliary skeletal representation was not consistently available in this range, which is expected due to the reduced spatial resolution of the human figures in thermal images. Three representative long-range cases were analyzed. At approximately 370 m, the right human was classified as outside the track zone, while the other two humans were classified as being inside the track zone and displayed with the ON RAIL warning status. The estimated distances were 346.6 m, 336.6 m, and 340.9 m, with confidence scores of 74%, 71%, and 75% (Figure 6a). At approximately 300 m, the left human was classified as being outside the track zone, while the other two remained inside the track zone and were displayed with the ON RAIL warning status; the estimated distances were 282.7 m, 283.0 m, and 281.0 m, with confidence scores of 76%, 72%, and 78% (Figure 6b). At approximately 250 m, all three humans were classified as being inside the track zone and were displayed with the ON RAIL warning status. The estimated distances were 225.0 m, 227.1 m, and 226.6 m, with confidence scores of 71%, 71%, and 75% (Figure 6c).

**Figure 7:**
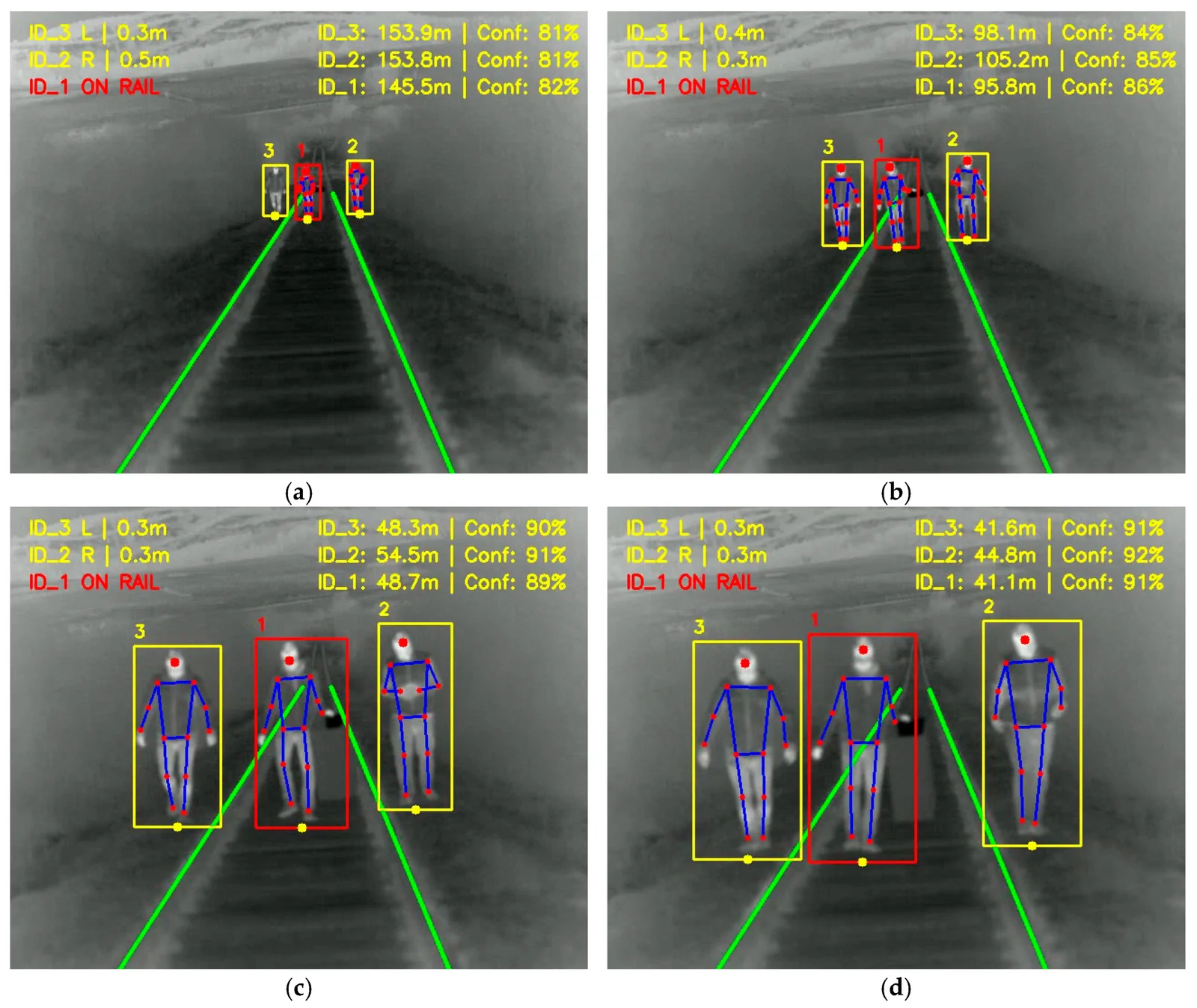
Results in three-human dynamic scenario in the range of approximately 200–40 m. The three humans are shown at measured reference distances of approximately (**a**) 150 m, (**b**) 100 m, (**c**) 50 m, and (**d**) 40 m from the thermal camera. The displayed outputs include rail track detection (marked in green), bounding boxes, identity labels (marked in yellow), track zone status with position of detected human with respect to the rail lines (L—left, R—right) and estimated lateral distance from the nearest rail line when the detected human is outside the track zone (marked in yellow), confidence scores (marked in yellow), estimated camera-to-human distances (marked in yellow), auxiliary skeletal representations (marked in blue links with red keypoints). Yellow bounding boxes and yellow track zone statuses indicate humans classified as being outside the track zone, whereas red bounding boxes indicate humans classified as being inside the track zone with displayed red ON RAIL track zone status.

After the humans approached to within approximately 200 m of the camera, the auxiliary skeletal representation became available together with bounding boxes, distance estimates, confidence scores, and track zone classification. In the analyzed cases from approximately 200 m to 40 m, the middle human was correctly classified as being inside the track zone and displayed with the ON RAIL warning status, while the other two humans were classified as outside the track zone. At approximately 200 m, the estimated distances were 196.5 m, 203.7 m, and 203.9 m, with confidence scores of 75%, 80%, and 81% (Figure 6d). At approximately 150 m, the estimated distances were 145.5 m, 153.9 m, 153.9 m, with confidence scores of 82%, 81%, and 81% (Figure 7a). At approximately 100 m, the estimated distances were 95.8 m, 105.2 m, and 98.1 m, with confidence scores of 86%, 85%, and 84% (Figure 7b). At approximately 50 m, the estimated distances were 48.7 m, 54.5 m, and 48.3 m, with confidence scores of 89%, 91%, and 90% (Figure 7c). Finally, at approximately 40 m, the estimated distances were 41.1 m, 44.8 m, and 41.6 m, with confidence scores of 91%, 92%, and 91% (Figure 7d).

Overall, the results indicate that the proposed framework can simultaneously detect and localize three humans, preserve interpretable bounding-box outputs, estimate camera-to-human distance, and classify the track zone status in a multi-human dynamic railway scenario. The system maintained functional detection even at long-range distances above 200 m, although the auxiliary skeletal representation became consistently available only after the humans approached approximately 200 m. The distance estimation error was larger in the long-range part of the sequence, with mean relative errors of approximately 7.7%, 5.9%, and 9.5% at around 370 m, 300 m, and 250 m, respectively. In contrast, after humans approached 200 m or less, the mean relative error generally decreased, ranging from approximately 1.9% to 6.3% across the analyzed short- and medium-range positions. This is consistent with the expected distance-dependent behavior of the rail track-guided estimation method: at larger distances, the apparent rail track width becomes smaller, so small pixel-level variations in rail line fitting and bounding box localization have a stronger influence on the final distance estimate. The confidence scores also increased as the humans approached the camera, from approximately 71–78% in the long-range cases to approximately 89–92% at 40–50 m, indicating improved detector certainty at shorter distances. Therefore, this scenario supports the feasibility of the proposed approach for multi-human thermal railway monitoring, while also showing that skeleton availability and distance estimation accuracy are strongly distance dependent.

**Figure 8:**
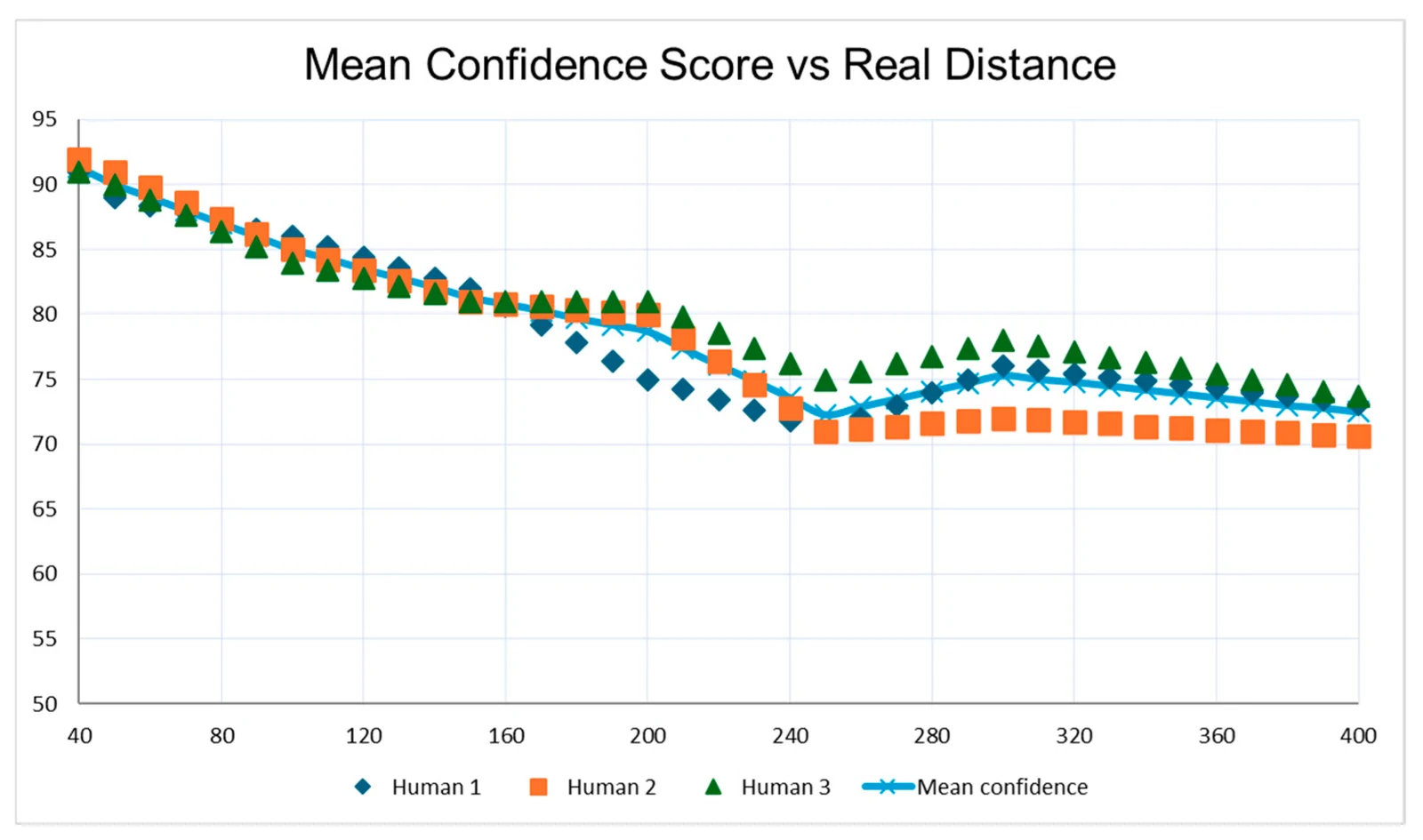
Mean Confidence Score vs. Real Distance.

The confidence score trend is consistent with the distance-dependent behavior observed in the previous scenario-based results (Figure 8). At larger measured camera-to-human distances, particularly in the long-range part of the sequence, the detector produced lower confidence values. This behavior is consistent with the nature of thermal imagery, where distant humans appear as small and less detailed thermal regions, and is expected because the three humans occupied fewer pixels in the thermal image and their body contours were less clearly visible. As the humans approached the camera, the confidence scores increased, reaching higher and more stable values in the medium- and short-range intervals. The individual confidence values for the three humans follow the same general trend and remain relatively close to the mean confidence curve, indicating consistent detector behavior in the analyzed multi-human scenario. Therefore, the confidence-score trend indicates that the proposed system is more reliable in medium- and short-range detection, while long-range detection remains feasible but more uncertain.

**Figure 9:**
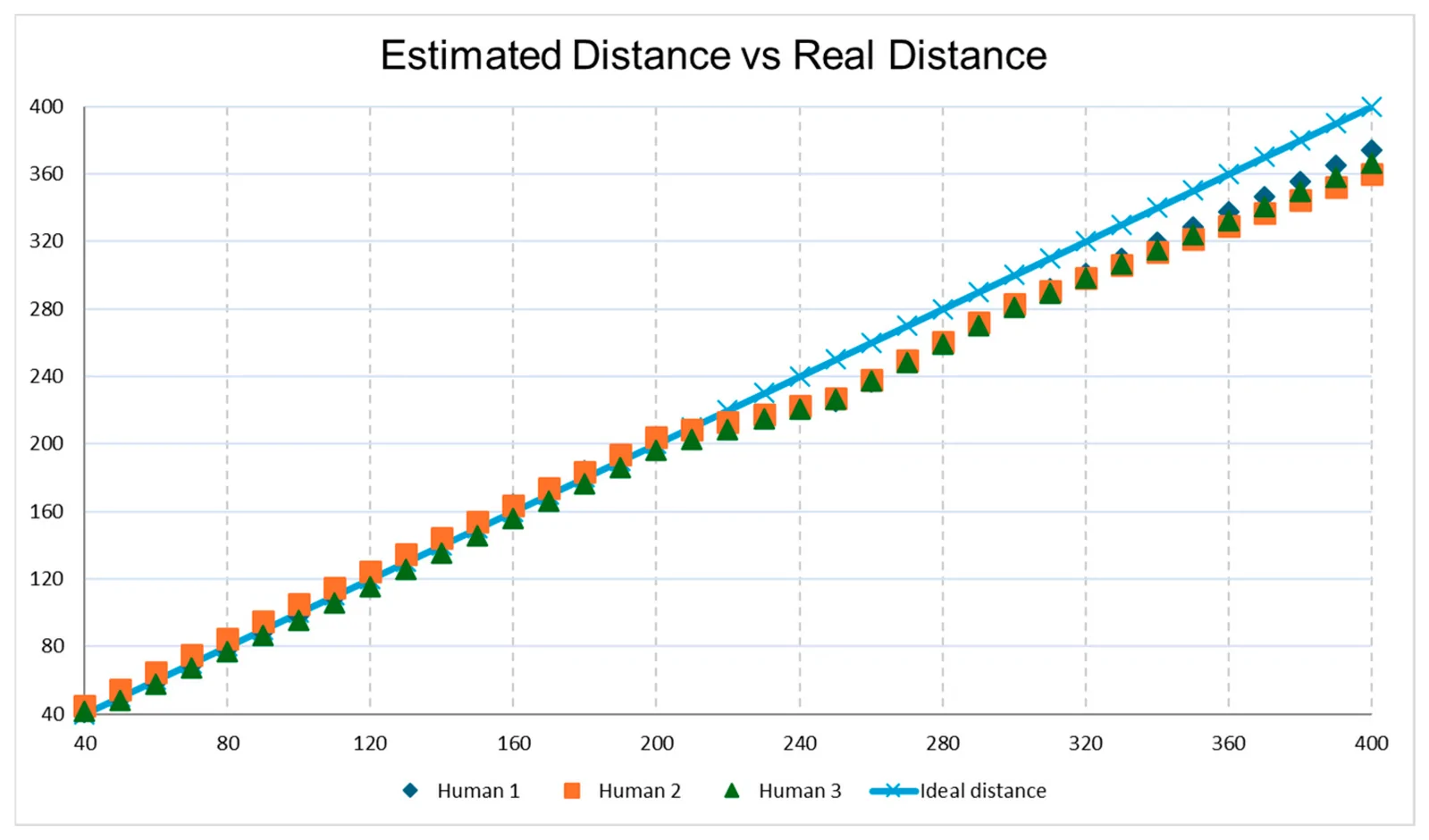
Estimated Distance vs. Real Distance.

The proposed rail track guided distance estimation method follows the real distance trend over the analyzed range (Figure 9). The estimated values for all three humans are generally close to the real distance, indicating that the method can consistently represent the change in camera-to-human distance as the humans move toward the camera. The results showed that estimation accuracy is higher in the medium- and short-range intervals, where the estimated distances are closer to the real distances and the dispersion between humans is lower. In the long-range part of the sequence, the estimated distances showed larger deviations from the real distance, which is expected because the apparent rail track width becomes smaller and the estimation becomes more sensitive to small pixel-level variations in rail line fitting and bounding box localization. Overall, the results confirm that the proposed method is able to capture the general distance trend for multiple humans, while also showing that the accuracy decreases as the real distance increases.

The quantitative evaluation of the proposed distance estimation was evaluated using calculation of MSE, MAE, RMSE and R^2^ score over the distance range from 400 m to 40 m (Table 5). The obtained results show that the estimated distances follow the real distance trend over the complete analyzed range, with a high R^2^ score indicating strong agreement between real and estimated values. However, the error metrics are higher than those obtained in the shorter distance range, which confirms that long-range distance estimation is more sensitive to small pixel-level variations in rail line fitting and bounding-box localization. This behavior is expected because the apparent rail track width decreases at larger distances, making the inverse-perspective estimation less stable. Overall, the results indicate that the proposed method can provide consistent distance estimation from long-range to short-range conditions, while showing improved accuracy as the detected humans approach the camera.

Previous homography-based railway distance-estimation studies reported low distance-estimation errors under calibrated conditions, including estimated distances of 49 m, 152 m, 295 m, and 491 m for real distances of 50 m, 150 m, 300 m, and 500 m, respectively, with a maximum reported error of approximately 2% [7]. The FuzzyH method was evaluated over longer distances, approximately from 400 m to 900 m [33]. Compared with these approaches, the present study does not focus solely on distance estimation, but integrates thermal human detection, auxiliary skeletal representation, identity-preserving tracking, temporal refinement, track zone classification, and rail track-guided distance estimation in a single thermal-only framework. Therefore, the comparison is interpreted qualitatively rather than as a direct benchmark, since the methods differ in calibration procedure, input assumptions, operating range, and evaluated system components. This comparison highlights that the proposed framework provides an integrated monitoring output, while the distance-estimation accuracy remains dependent on rail line fitting quality, bounding-box localization, and the validity of the straight-track ground-plane assumption.

To further evaluate the reliability of the track zone classification procedure, an additional frame-level validation was performed. A total of 200 annotated human samples extracted from video frames were manually inspected and assigned to one of two classes: inside the track zone, corresponding to the ON RAIL class, or outside the track zone, corresponding OFF RAIL class (Table 6). The manually assigned labels were used as reference labels and compared with the automatically estimated track zone status obtained using the proposed geometric classification procedure. The resulting confusion matrix is presented in Table 6. The evaluated set contained 90 ON RAIL and 110 OFF RAIL samples. The samples included different human positions with respect to the fitted rail lines, as well as representative near-boundary cases in which classification is more challenging. The samples were selected from the experimental video sequences used for framework evaluation and were independent of the reference frames used for distance calibration. To reduce temporal redundancy, the samples were taken from different parts of the sequences and were not selected as a continuous block of consecutive, highly similar frames. This selection ensured that the validation set included both clear ON RAIL/OFF RAIL cases and near-boundary cases.

The obtained results indicate that the proposed track zone classification procedure correctly classified 92.0% of the manually inspected samples (Table 7). The recall of 90.0% shows that most safety-critical on rail cases were correctly identified, while the precision of 92.0% indicates that most generated on rail warnings corresponded to actual inside-track-zone situations. The F1-score of 91.0% indicates balanced performance between missed on rail cases and false on rail warnings. Most observed classification errors occurred in near-boundary cases, where the bottom-center point of the human bounding box was close to one of the rail boundaries, making the result sensitive to small localization or rail line fitting errors.

The proposed framework was developed and evaluated for detecting humans located on or in the immediate vicinity of the rail track, with particular emphasis on safety-critical inside-track zone situations. The distance-estimation and track zone classification procedures rely on the visibility of the rail track geometry, a fixed and centrally positioned thermal camera, a known railway gauge, and the assumption that the detected human is located on or close to the railway ground plane. Therefore, the reliability of the estimated distance and track zone status depends on the quality of human bounding-box localization, rail line fitting, and the visibility of both rail lines. The available thermal images contain less structural and anatomical detail than high-resolution RGB images, which is a hardware-related constraint associated with the thermal sensor resolution, optics, and acquisition distance. Finally, the relatively limited size of the annotated thermal datasets constrains broader generalization, and further validation on larger and more diverse datasets is required.

The results also indicate a distance-dependent degradation in the performance of the proposed framework. At distances above approximately 200 m, human detection and distance estimation remained possible in the analyzed scenarios, but the auxiliary skeletal representation became less consistently available because distant humans occupied only a small number of pixels in the thermal image. In this range, reduced spatial resolution and weaker thermal contrast increase the sensitivity of bounding-box localization, rail line fitting, and distance estimation. Therefore, the system output at long range should be interpreted primarily as early warning information, while more reliable auxiliary skeletal representation and more stable spatial interpretation are obtained when the detected human approaches the medium- and short-range intervals below approximately 200 m.

Although model inference was performed on Google Colab using an NVIDIA T4 GPU, the end-to-end processing speed of the complete framework was not systematically evaluated in this study. The current implementation was used for experimental offline analysis and was not optimized for real-time deployment. Therefore, real-time performance is not claimed. A detailed latency analysis, including human detection, auxiliary pose extraction, rail track segmentation, ByteTrack association, Kalman-based temporal refinement, track zone classification, and distance estimation on target deployment hardware, will be addressed in future work.

## 5. Conclusions

This paper presented a thermal-only deep learning-based railway monitoring framework for human detection, auxiliary skeletal representation extraction, identity-preserving tracking, temporal refinement, track zone classification, and rail track-guided distance estimation under low-visibility conditions. The proposed approach integrates YOLO-based human and rail track detection with a pretrained YOLO pose model, ByteTrack-based identity association, and Kalman-based temporal refinement in order to provide stable and interpretable outputs in real railway environments.

The experimental results indicate that the proposed framework can detect and localize humans in both controlled and dynamic railway scenarios, including multi-human cases over a wide distance range. The system provides bounding boxes, tracking identities, confidence scores, estimated camera-to-human distances, and track zone status, while the auxiliary skeletal representation becomes consistently available primarily in the medium- and short-range intervals where sufficient image detail is available. The additional sample-level validation of track zone classification showed an accuracy of 92.0%, precision of 92.0%, recall of 90.0%, and F1-score of 91.0%, indicating reliable identification of safety-critical on rail cases in the analyzed scenarios. In addition, Kalman-based temporal refinement reduced inter-frame keypoint localization jitter in the analyzed stationary sequence, confirming its contribution to the temporal stability of the auxiliary skeletal representation. The strongest stabilization was observed for the head keypoint of human ID 1, where the jitter decreased by 54.9%, while the average full-body jitter across all 13 keypoints decreased by 24.6% for human ID 1 and by 24.1% for human ID 2. A stationary segment was selected because it allows the temporal jitter introduced by frame-to-frame keypoint localization variability to be isolated from true physical motion. In dynamic sequences, inter-frame displacement contains both real human motion and localization jitter; therefore, evaluation of Kalman filtering under motion would require frame-level ground-truth trajectories and is left for future work. The results also showed the expected distance-dependent behavior of thermal imaging. Confidence scores increased as humans approaching the camera, while long-range auxiliary skeletal representation availability and distance estimation became more sensitive to reduced spatial detail, rail line fitting variations, and bounding-box localization errors. Compared with approaches based solely on classical image processing or geometry-driven procedures, the proposed framework provides a more integrated monitoring output within the analyzed scenarios, primarily by combining human detection, auxiliary skeletal representation, identity-preserving tracking, track zone classification, distance estimation, and temporal refinement. Furthermore, the proposed approach estimates camera-to-human distance from the apparent rail track width at the vertical position of the detected human, using the known railway gauge. This allows the system to provide distance estimates without requiring an additional range sensor, although the accuracy remains dependent on rail visibility, rail line fitting quality, bounding-box localization, and the validity of the straight-track ground-plane assumption.

Despite these advantages, the system performance degrades at distances beyond approximately 200 m, where the auxiliary skeletal representation becomes less consistently available and distance-estimation error increases due to limited spatial resolution and reduced spatial detail. Therefore, long-range detections should be interpreted primarily as early warning information, while more reliable skeletal visualization and spatial interpretation are obtained in medium- and short-range intervals. Additionally, the relatively limited annotated thermal datasets constrain broader generalization. Future work will focus on extending the datasets, improving long-range performance through enhanced feature learning and multi-scale modeling, and exploring multimodal fusion approaches combining thermal, RGB, and additional sensor data to further increase robustness and accuracy in real-world railway applications, and performing end-to-end latency evaluation and optimization on target deployment hardware.

## Figures and Tables

**Table 1:** Reference distance and rail track width.

Reference Distance $D_{ref}$ [m]	Rail Width $w_{px}$ [pixels]	Individual $K_{cal}$
30 m	311 px	6502
90 m	103 px	6460
150 m	62 px	6481

**Table 2:** Results in static and quasi-static scenarios.

Human Status/No. of Humans	Real Distance [m]	Estimated Distance [m]	Error [m]	Error [%]	Confidence [%]
Stationary/2	50	52.9	2.9	5.8	90
Stationary/2	100	98.4	1.6	1.6	84
Moving/2	50	52.9	2.9	5.8	88
Moving/2	100	98.4	1.6	1.6	85

**Table 3:** Results of distance estimation and confidence in dynamic zig-zag short-range scenario.

Human Status/ No. Of Humans	Real Distance [m]	Estimated Distance [m]	Mean Absolute Error [m]	Mean Absolute Error [%]	Confidence [%]
Stationary/2	150	146.1–147.6	3.15	2.1	79–80
Moving/2	90	83.5	6.5	7.2	82
Moving/2	70	68.2	1.8	2.6	84
Moving/2	50	49.1	0.9	1.8	84
Moving/2	40	39.1	0.9	2.3	84

**Table 4:** Representative distance-estimation checkpoints in the range of approximately 400–40 m.

Human Status/ No. of Humans	Real Distance [m]	Estimated Distance [m]	Mean Absolute Error [m]	Mean Absolute Error [%]	Confidence [%]
Moving/3	~400	374.0, 359.6, 366.6	33.3	8.3	73, 71, 74
Moving/3	~370	346.6, 336.6, 340.9	28.6	7.7	74, 71, 75
Moving/3	~300	282.7, 283.0, 281.0	17.7	5.9	76, 72, 78
Moving/3	~250	225.0, 227.1, 226.6	23.8	9.5	71, 71, 75
Moving/3	~200	203.9, 203.7, 196.5	3.7	1.9	75, 80, 81
Moving/3	~150	153.9, 153.8, 145.5	4.1	2.7	82, 81, 81
Moving/3	~100	98.1, 105.2, 95.8	3.8	3.8	86, 85, 84
Moving/3	~50	48.7, 54.5, 48.3	2.5	5.0	89, 91, 90
Moving/3	~40	41.1, 44.8, 41.6	2.5	6.3	91, 92, 91

**Table 5:** MSE, MAE, RMSE and R2 score for range from 400 to 40 m.

	MSE	MAE	RMSE	R^2^ Score
All humans	290.09	13.26	17.03	0.9746
Human 1	226.96	11.61	15.07	0.9801
Human 2	341.48	14.36	18.48	0.9700
Human 3	301.83	13.81	17.37	0.9735

**Table 6:** Confusion matrix for frame-level track zone classification.

	Predicted ON RAIL	Predicted OFF RAIL	Total
Reference ON RAIL	81	99	90
Reference OFF RAIL	7	103	110
Total	88	112	200

**Table 7:** Quantitative evaluation of track zone classification.

Metric	Value
Accuracy	92.0%
Precision	92.0%
Recall	90.0%
F1-score	91.0%

## Data Availability

The original contributions presented in this study are included in the article. Further inquiries can be directed to the corresponding author.
